# CMR-based assessment of long-term effects of tafamidis in patients with cardiac transthyretin amyloidosis

**DOI:** 10.1007/s00392-025-02691-8

**Published:** 2025-10-27

**Authors:** Alexandru Zlibut, Michael Bietenbeck, Nuriye Akyol, Volker Vehof, Redouane Bouras, Khuraman Isgandarova, Claudia Meier, Maria Theofanidou, Philipp Stalling, Ali Yilmaz

**Affiliations:** Department of Cardiology I, Division of Cardiovascular Imaging, University Hospital Münster, Von-Esmarch-Str. 48, 48149 Münster, Germany

**Keywords:** Cardiac amyloidosis, CMR, Tafamidis, ATTR

## Abstract

**Objectives and background:**

The aim of the study was to evaluate the long-term effects of tafamidis on cardiac disease progression beyond 12 months of treatment by performing (among others) serial multi-parametric cardiovascular magnetic resonance (CMR) studies in patients with transthyretin (ATTR) cardiac amyloidosis (CA) cardiomyopathy (ATTR-CM).

**Methods:**

Patients with confirmed ATTR-CM (*N* = 56) were divided into two groups: in the larger group A (*N* = 39; 95% male), treatment with tafamidis 61 mg once daily was initiated after the first CMR study, whereas group B (*N* = 17; 76% male) comprised ATTR-CM patients who did not receive tafamidis. The observational follow-up period lasted 27 ± 6 months. During this period, patients underwent two multi-parametric CMR studies at our institution as part of a routine clinical observation pipeline.

**Results:**

Clinical symptoms assessed by the NYHA class showed a slight, however, significant increase in both groups. NT-proBNP levels substantially increased in both groups at follow-up, however, with a significantly higher increase in the tafamidis-naïve group B (*p* = 0.014). LV systolic function, defined by LV-EF and 3D global longitudinal peak strain, significantly worsened in both groups at follow-up (54% to 48% *p* < 0.001 vs 56% to 46%, *p* < 0.001; −7.4 to −5.3, *p* < 0.001 vs −8.8 to −4.8, *p* < 0.001). However, the tafamidis-naïve group B experienced a substantially higher impairment of both parameters when compared to group A (∆p = 0.008 and ∆p = 0.003, respectively). LV wall thickness considerably increased in both groups at follow-up, however, with a significantly higher increase in the tafamidis-naïve group B (from 18.2 mm to 21.1 mm at follow-up, *p* < 0.001) compared to the tafamidis-treated group A (from 18.5 mm to 19.2 mm, p = 0.012; ∆*p* < 0.001). Both global native T1 and global ECV values were significantly elevated in both groups—at baseline and at follow-up—with a significant increase in both groups during follow-up. However, a substantially higher increase in global ECV was observed in the tafamidis-naïve group B compared to the tafamidis-treated group A (group A: 51% to 57%, *p* < 0.001; group B: 50% to 67%, *p* < 0.001; ∆p < 0.001).

**Conclusion:**

Substantial worsening of clinical symptoms, serum biomarkers, and imaging parameters occurred in both tafamidis-treated and tafamidis-naïve ATTR-CM patients within a follow-up period of approximately 2 years. However, the “extent of worsening” is significantly lower in the tafamidis-treated compared to the tafamidis-naïve patients.

**Graphical abstract:**

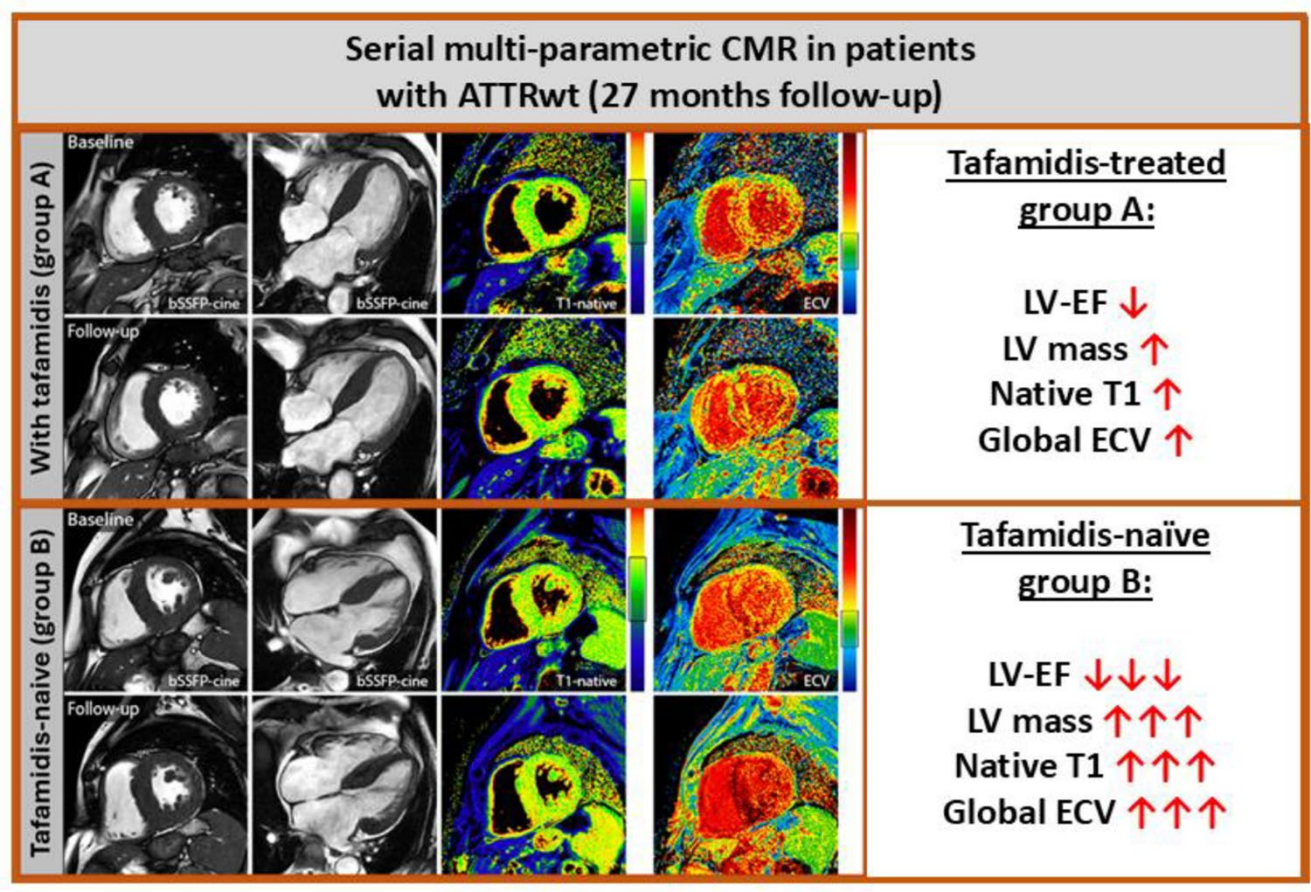

## Introduction

Cardiac amyloidosis (CA) is an infiltrative heart disease characterized by the accumulation of misfolded transthyretin proteins within the extracellular space of the myocardium [1, 2]. The most common forms of CA are a) immunoglobulin light chain (AL) and b) transthyretin amyloidosis (ATTR) that in turn comprises two subtypes: a hereditary form (ATTRv) caused by the presence of a TTR gene mutation and a wild-type form (ATTRwt) caused by age-related instability of wild-type TTR [3, 4]. ATTR-cardiomyopathy (ATTR-CM) is characterized by progressive heart failure, supraventricular and ventricular arrhythmias, and cardiovascular death [5].

Currently, non-invasive imaging techniques, particularly cardiovascular magnetic resonance (CMR) and bone scintigraphy, are used to detect CA [6, 7]. Moreover, CMR methods for myocardial tissue characterization, such as late gadolinium enhancement (LGE), pre- and post-contrast T1-mapping with extracellular volume fraction (ECV) quantification, enable precise assessment regarding cardiac disease extent and progression [5, 7, 8].

Currently, tafamidis—a TTR stabilizer—is the only approved specific drug to treat ATTR-CM. In the well-known ATTR-ACT trial, tafamidis treatment significantly reduced all-cause mortality, hospitalizations for heart failure, and increased quality of life [9]**.** Furthermore, a post hoc analysis of this trial demonstrated that tafamidis treatment also prevented the occurrence of LV systolic and diastolic dysfunction at 30 months follow-up [10].

So far, only a few single-center studies evaluated the treatment effects of tafamidis using a multi-parametric CMR approach for monitoring cardiac disease progression, as well as response to therapy [11–13]. Available studies indicate that tafamidis treatment may stop or at least slow down cardiac disease progression after a treatment period of 12 months. However, long-term studies beyond 12 months of follow-up and with careful assessment of cardiac changes based on multi-parametric CMR have not been published yet.

The aim of the study was to evaluate the long-term effects of tafamidis on cardiac disease progression beyond 12 months of follow-up by performing (among others) serial multi-parametric CMR studies in patients with ATTR-CM.

## Methods

### Patient characteristics and study design

A total of 56 patients (89% male) diagnosed with either histologically confirmed ATTR amyloidosis and/or positive results on both bone scintigraphy and CMR (in addition to negative monoclonal protein studies) were included in this retrospective single-center study.

Exclusion criteria were the presence of monoclonal gammopathy and/or other forms of cardiac amyloidosis including hereditary ATTR (ATTRv) that was ruled out by genetic testing. Additionally, individuals newly diagnosed with cancer, advanced renal disease, new-onset atrial fibrillation, cardiac implantable electronic devices causing significant image artifacts, or those who reached 85 years of age were excluded from the study.

The observational follow-up period lasted 27 ± 6 months. During this period, patients underwent at least two multi-parametric CMR studies at our institution as part of a highly standardized routine clinical observation pipeline. The National Amyloidosis Centre (NAC) staging score was used to assess the ATTR disease stage. All visits included assessment of cardiac and renal biomarkers as well as evaluation of heart failure symptoms based on NYHA class.

Patients with confirmed ATTR-CM were divided into two groups based on the decision whether to start a disease-specific tafamidis therapy: in the larger group A (*N* = 39; 95% male), treatment with tafamidis 61 mg once daily was initiated after the first CMR study, whereas group B (*N* = 17; 76% male) comprised ATTR-CM patients who did not receive tafamidis, mainly due to personal choice and/or medicolegal reasons. Gene-silencing agents such as patisiran or inotersen were not used prior to or during the follow-up of this study in any patient included.

In principle, withholding tafamidis in patients with ATTR-CM is ethically not justifiable—unless there are relevant contraindications or medicolegal issues. Since we have established standardized procedures regarding diagnosis and follow-up of ATTR-CM at our center, we were able to collect standardized and robust “cardiac” data in both patients with and without tafamidis.

### Ethics approval and consent to participate

Written informed consent was obtained from every patient prior to study inclusion. The study protocol was in accordance with the ethical guidelines of the 1975 Declaration of Helsinki and with the laws and regulations of Germany. The protocol was approved by the local ethics committee (Ethikkommission der Ärztekammer Westfalen-Lippe) of the University Hospital Muenster, Germany (ID 2019–437-f-S).

### CMR acquisition and analysis

Two 1.5-T systems were used for CMR imaging (Ingenia and Ingenia Ambition X, Philips Healthcare, Best, The Netherlands), utilizing protocols and settings suggested by the Society for Cardiovascular Magnetic Resonance (SCMR): cine and late gadolinium enhancement acquisitions were conducted in standard short- and long-axis views. A modified Look–Locker inversion recovery T1-mapping sequence was employed in basal, mid, and apical short axes—both before and approximately 15 min following the administration of 0.15 mmol/kg gadolinium (Gadobutrol) to determine the measures of native T1 and extracellular volume fraction (ECV). Motion-corrected and segmented ECV maps were generated from the native and post-contrast segmented T1-maps, using the patient’s hematocrit level.

For the assessment of global LV deformation, three-dimensional (3D) LV global longitudinal strain (LV-GLS) derived from feature tracking (FT) was obtained using a validated algorithm integrated in the analysis software. Relative apical longitudinal strain (LS) was calculated based on the following equation: average apical LS/(average basal LS + mid LS).

Analysis and postprocessing of the acquired images were performed using cvi42 (version 6.0.0, Circle Cardiovascular Imaging, Calgary, Alberta, Canada), as previously outlined [11].

### Statistical analysis

The Shapiro–Wilk test was used to assess normal distribution of parameters. All variables were non-normally distributed. To evaluate potential differences between groups, Mann–Whitney *U* tests were used, while Wilcoxon signed-rank tests were conducted looking for temporal changes in a single patient cohort. Statistical analysis was performed with SPSS (version 29.0, IBM Corp., Armonk, NY). Data are presented as median (interquartile range) and a p value < 0.05 was considered statistically significant.

## Results

### Baseline characteristics of the study population

Baseline clinical characteristics as well as serum and imaging parameters are summarized in Table 1. Patients included in the final analysis (*n* = 56) were divided into two groups: group A (with tafamidis; *N* = 39) and group B (without tafamidis; *N* = 17). There were no relevant differences in the clinical characteristics between the two groups, sharing similar values in terms of mean age (*p* = 0.77), BMI (*p* = 0.96), eGFR (*p* = 0.65), NAC stage (*p* = 0.31), or NYHA class (*p* = 0.23). NT-proBNP levels were considerably higher in the tafamidis-naïve group B, however, without reaching statistical significance (*p* = 0.06).

**Table 1:** Baseline patient characteristics

Parameter	Group A = with tafamidis	*n*	Group B = tafamidis-naïve	*n*	*p* value
Age (years)	79 (76–82)	39	80 (76–81)	17	0.77
Males/females	37/2	39	13/4	17	**0.041**
BMI (kg/m^2^)	26 (24–29)	39	26 (24–29)	17	0.96
eGFR (CKD-EPI) (ml/min/1.73 m^2^)	58 (47–67)	39	60 (54–68)	17	0.65
NYHA class	2 (1.0–2.0)	39	2.0 (2.0–3.0)	17	0.23
NT-proBNP (pg/ml)	1,421 (887–2,164)	39	2,250 (1,207–4,373)	17	0.06
NAC (National Amyloidosis Centre) staging score					
Stage I	24	39	8	17	0.31
Stage II	10		5		
Stage III	6		4		
Major CMR findings					
LV-EF (%)	54 (50–59)	39	56 (49–61)	17	0.19
LV-EDVi (ml/m^2^)	88 (76–96)	39	85 (74–96)	17	0.48
LV mass index (g/m^2^)	92 (78–110)	39	87 (70–118)	17	0.75
Max. LV thickness (mm)	19 (17–20)	39	18 (16–22.5)	17	0.48
RV-EF (%)	52 (50–56)	39	49 (45–50)	17	**0.017**
RV-EDVi (ml/m^2^)	82 (72–98)	39	95 (78–103)	17	0.18
3D global longitudinal Peak strain (%)	−7.4 (−8.9 to −5.8)	37	−8.8 (−9.8 to −7.5)	17	**0.016**
Apical/(basal + mid) strain ratio (3D), n	0.81 (0.72–0.98)	37	0.81 (0.80–0.91)	17	0.39
Global native T1 [950–1050 ms]	1,089 (1,055–1,108)	39	1,108 (1,083–1,131)	17	0.06
Basal septal native T1 [950–1050 ms]	1,083 (1,061–1,099)	39	1,111 (1,090–1,120)	17	**0.006**
Global ECV [25–31%]	51 (47–57)	39	50 (42–53)	17	0.46

All data are given as median (interquartile range). *BMI* body mass index; *eGFR* *(CKD-EPI)* estimated glomerular filtration rate according to Chronic Kidney Disease Epidemiology Collaboration, *NYHA* New York Heart Association, *NT-proBNP* N-terminal pro brain natriuretic peptides, *CMR* cardiovascular magnetic resonance, *LV* left ventricle, *RV* right ventricle, *EF* ejection fraction, *EDVi* end-diastolic volume index, *ECV* extracellular volume fraction, *p* < 0.05 is considered as significant (bold); *n* = respective number of patients with available parameters

With respect to CMR parameters, the two groups showed overall similar imaging findings at baseline regarding both function and myocardial tissue characterization: LV systolic function (*p* = 0.19), LV-EDVi (*p* = 0.48), LV mass (*p* = 0.75), maximal LV wall thickness (*p* = 0.48), global native T1 (*p* = 0.06), and ECV (*p* = 0.46).

Additionally, there was no need for discontinuation of tafamidis therapy in group A, being well tolerated without any significant adverse reactions by all patients.

### Longitudinal assessment of clinical and serum parameters

Clinical symptoms assessed by the NYHA class showed a slight, however, significant increase in both groups (Tables 1, 2), with a trend to a more severe increase in symptoms in the tafamidis-naïve group B at the end of the follow-up period.

**Table 2:** Biomarkers and CMR changes over a mean follow-up of 27 months

Parameter	Group A = with tafamidis (*N* = 39)			Group B = tafamidis-naïve (*N* = 17)			∆ p value
	Baseline	Follow-up	*p*-value	Baseline	Follow-up	*p*-value	
NYHA	1.9 (1.0–2.1)	2.4 (2.0–3.0)	**0.001**	2.0 (2.0–3.0)	3.0 (2.5–3.0)	**0.002**	0.46
NT-proBNP (pg/ml)	1,848 (887–2,164)	2,202 (1,328–4,075)	** < 0.001**	2,250 (1,207–4,373)	3,717 (1,824–8,709)	** < 0.001**	0.05
Troponin-T hs (ng/l)	38 (29–48)	37 (29–62)	0.06	49 (32–74)	60 (53–107)	**0.001**	**0.014**
Major CMR findings							
LV-EF (%)	54 (50–59)	48 (43–52)	** < 0.001**	56 (49–61)	46 (40–51)	** < 0.001**	**0.008**
LV-EDVi (ml/m^2^)	88 (76–96)	92 (83–106)	**0.009**	85 (74–96)	85 (75–94)	0.64	0.35
LV mass index (g/m^2^)	92 (78–110)	96 (79–111)	0.14	87 (70–118)	102 (80–127)	** < 0.001**	**0.031**
Max. LV thickness (mm)	18.5 (17.0–20.0)	19.2 (18.0–20.0)	**0.012**	18.2 (15.5–22.5)	21.1 (19.0–23.5)	** < 0.001**	** < 0.001**
RV-EF (%)	52 (50–56)	47 (42–52)	** < 0.001**	49 (45–50)	44 (35–46)	** < 0.001**	0.96
RV-EDVi (ml/m^2^)	82 (72–98)	89 (75–101)	0.054	95 (78–103)	96 (68–119)	0.32	0.71
3D global longitudinal peak strain (%)	−7.4 (−8.9 to −5.8)	−5.3 (−5.9 to −3.4)	** < 0.001**	−8.8 (−9.8 to −7.5)	−4.8 (−5.8 – −4.0)	** < 0.001**	**0.003**
Apical/(basal + mid) strain ratio (3D), n	0.81 (0.72–0.98)	0.99 (0.94–1.15)	** < 0.001**	0.81 (0.80–0.91)	0.99 (0.98–1.27)	** < 0.001**	0.53
Global native T1 [950–1050 ms]	1,085 (1,055–1,108)	1,097 (1,064–1,135)	**0.018**	1,108 (1,083–1,131)	1,129 (1,104–1,134)	**0.036**	0.72
Basal septal native T1 [950–1050 ms]	1,083 (1,061–1,099)	1,095 (1,061–1,130)	**0.039**	1,111 (1,090–1,120)	1,122 (1,087–1,140)	0.18	0.78
Global ECV [25–31%]	51 (47–57)	57 (52–67)	** < 0.001**	50 (42–53)	67 (58–71)	** < 0.001**	** < 0.001**

All data are given as median (interquartile range), if not mentioned otherwise. Units are mentioned in small brackets (). Normal range of values are mentioned in large brackets []. *CMR* cardiovascular magnetic resonance, *LV* left ventricle, *RV* right ventricle, *EF* ejection fraction, *EDVi* end-diastolic volume index, *ECV*—extracellular volume fraction, *p*—significance between baseline and follow-up within a group, ***∆****p*—significance between the changes among treated and untreated patients in the respective parameters, *p* < 0.05 is considered as significant (bold)

Regarding serum parameters, NT-proBNP levels substantially increased in both groups at follow-up, however, with a significantly higher increase in the tafamidis-naïve group B (*p* = 0.014). In addition, tafamidis-naïve patients (group B) showed a considerable and significant increase in serum troponins that was not observed in the tafamidis-treated group A.

### Longitudinal assessment of CMR parameters

LV systolic function, defined by LV-EF and 3D global longitudinal peak strain, significantly worsened in both groups at follow-up (54% to 48% *p* < 0.001 vs 56% to 46%, *p* < 0.001; −7.4 to −5.3, *p* < 0.001 vs −8.8 to −4.8, p < 0.001). However, the tafamidis-naïve group B experienced a substantially higher impairment of both parameters when compared to group A (∆p = 0.008 and ∆p = 0.003, respectively) (Table 2).

In line with the aforementioned functional results, LV wall thickness considerably increased in both groups at follow-up, however, with a significantly higher increase in the tafamidis-naïve group B (from 18.2 mm to 21.1 mm at follow-up, *p* < 0.001) compared to the tafamidis-treated group (from 18.5 mm to 19.2 mm, *p* = 0.012; ∆p < 0.001). In addition, the tafamidis-naïve group B showed a substantial increase in LV mass index at follow-up (87 to 102 g/m^2^, *p* < 0.001) that was not observed in the tafamidis-treated group A (Table 2).

Regarding CMR-based myocardial tissue parameters, both global native T1 and global ECV values were significantly elevated in both groups—at baseline and at follow-up—with a significant increase in both groups during follow-up. However, a substantially higher increase in global ECV was observed in the tafamidis-naïve group B compared to the tafamidis-treated group A (group A: 51% to 57%, *p* < 0.001; group B: 50% to 67%, *p* < 0.001; ∆p < 0.001) (Table 2).

## Discussion

To the best of our knowledge, our present study is the first that monitored cardiac disease progression in patients with ATTR-CM, either receiving tafamidis (group A) or being tafamidis-naïve (group B) using multi-parametric CMR data for a long follow-up period of 27 ± 6 months. The present retrospective data do not only allow to carefully assess the natural cardiac disease progression in tafamidis-naïve ATTR-CM patients, but also enable to assess the effect of a long-term tafamidis treatment in such ATTR-CM patients. The major findings of the present study can be summarized as follows: 1) a substantial worsening of a) clinical symptoms, b) serum biomarkers, and c) imaging parameters was observed in tafamidis-naïve ATTR-CM patients within a follow-up period of approximately 2 years. 2) A similar worsening in a) clinical symptoms, b) serum biomarkers, and c) imaging parameters was also observed in ATTR-CM patients receiving tafamidis, but the “extent of worsening” was significantly lower in the tafamidis-treated group A compared to the tafamidis-naïve group B.

### Comparison of 12 months data vs. 27 months data of tafamidis treatment

**Fig. 1:**
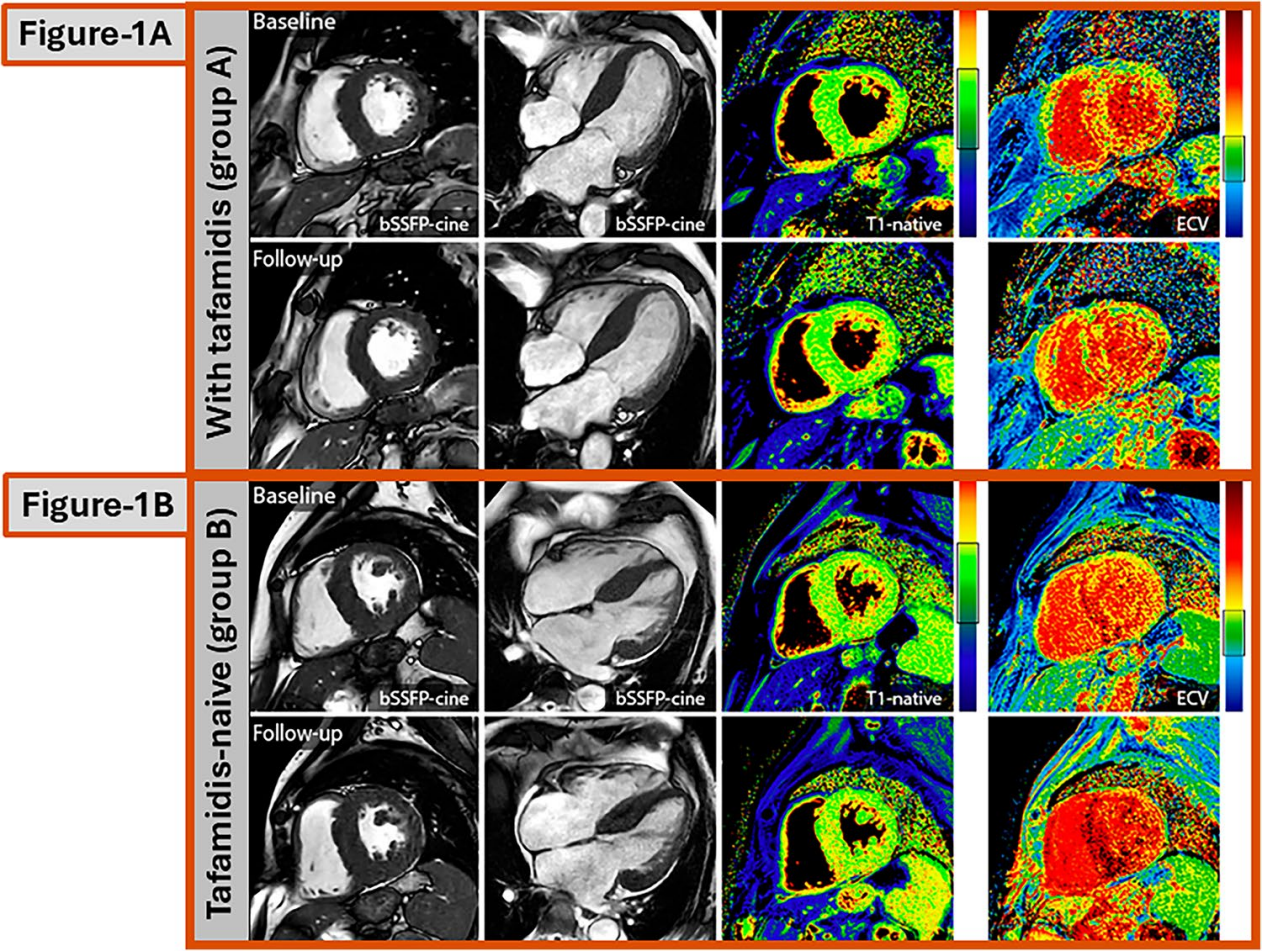
**A** Cardiovascular magnetic resonance (CMR) images of a tafamidis-treated patient from group A. Baseline and follow-up images are shown in the following order: short-axis cine images, long-axis cine images, native T1-maps, and ECV maps. In this tafamidis-treated patients, the following minor changes were observed during the study period of approximately 2 years: LV-EF = 59%–54%; LV mass = 95 g/m^2^–93 g/m^2^; native T1 = 1,117 ms–1,094 ms; global ECV = 65%–70%. **B** Cardiovascular magnetic resonance (CMR) images of a tafamidis-naïve patient from group B. Baseline and follow-up images are shown in the following order: short-axis cine images, long-axis cine images, native T1-maps, and ECV maps. In this tafamidis-naïve patients, the following substantial changes were observed during the study period of approximately 2 years: LV-EF = 62%–48%; LV mass = 108 g/m^2^–150 g/m^2^; native T1 = 1,094 ms–1,132 ms; global ECV = 50%–72%

In the past, our group had published similar clinical, biomarker, and imaging data that were collected in ATTR-CM patients within a follow-up period of only 12 months [11]. In that previous study, tafamidis therapy in patients with ATTR-CM did not “improve” cardiac disease status after 1 year of therapy, but substantially “stopped” cardiac disease progression compared to those patients with ATTR-CM who did not receive tafamidis. Importantly, a trend toward improvement in clinical symptoms based on NYHA class was also observed in patients with ATTR-CM receiving tafamidis in that prior study with a shorter follow-up time. In contrast, our present study with an extended follow-up time of 27 ± 6 months clearly shows that tafamidis treatment per se is not able to “stop” cardiac disease progression in ATTR-CM patients (neither regarding clinical symptoms or biomarkers nor regarding imaging parameters), but substantially “slows down” cardiac disease progression when compared to a comparable tafamidis-naïve group **(**Fig. 1A-B**)**.

### Long-term effect of tafamidis on CMR-based functional parameters

In our previous study with a follow-up time of only 12 months [11], an overall deterioration of LV-EF was observed only in the tafamidis-naïve group (57% to 51%, *p* = 0.003), but not in the tafamidis-treated group (51% to 51%, *p* = 0.75). In comparison, in the current study with a longer follow-up time of 27 months, we observed a significant decrease in LV-EF in both groups (54% to 48%, *p* < 0.001, in the tafamidis-treated group vs. 56% to 46%, *p* < 0.001, in the tafamidis-naïve group), however, with a more pronounced decrease in the tafamidis-naïve group (∆p = 0.008). In contrast, another TTR stabilizer, acoramidis, was recently shown to even improve LV-EF (51% to 56%) after 30 months of treatment [14]—indirectly suggesting a potentially superior effect of acoramidis compared to tafamidis.

At the same time, a significant increase in maximal LV wall thickness as well as in total LV mass was observed in both groups in our study—with significantly higher increases in the tafamidis-naïve group (∆p < 0.001 and ∆p = 0.031). Hence, the present data illustrate that LV mass substantially expands and LV systolic function starts to get impaired due to ongoing amyloid deposition in the myocardium in spite of an ongoing tafamidis treatment for a treatment period of approximately 2 years. In contrast, acoramidis was shown to prevent an increase in LV mass after 30 months of treatment [14]—again supporting the notion of a potentially superior effect of acoramidis compared to tafamidis. 

In our previous study with a follow-up time of only 12 months [11], longitudinal inter-group changes did not show relevant differences in feature-tracking based 3D-LV-GLS between the groups with and without tafamidis (∆p = 0.46). In comparison, in the current study with a longer follow-up time of 27 months, we observed a significant worsening of peak 3D-LV-GLS in both groups (−7.4% to −5.3% in the tafamidis-treated group vs. −8.8% to −4.8% in the tafamidis-naïve group), however, with a significantly more pronounced worsening in the tafamidis-naïve group (∆p = 0.003). The apical-sparing phenomenon was also assessed in the present study, but despite a significant longitudinal worsening in each group, we could not observe a substantial difference between the tafamidis-treated compared to the tafamidis-naïve group (∆p = 0.53). Hence, the present results are in line with recent studies that questioned the diagnostic yield and value of strain-based assessment of apical-sparing phenomenon in the context of cardiac amyloidosis [15].

### Long-term effect of tafamidis on CMR-based myocardial tissue parameters.

Recently, Duca et al. evaluated data from a prospective observational patient registry (in Austria) and were able to compare multi-parametric CMR data in tafamidis-naïve ATTR-CM patients and tafamidis-treated ones for a follow-up period of 12 months [13]. Numerically similar “deteriorations” in LV-EF, LV-EDV, and RV-EDV were observed in both groups in that study. However, ECV (measured only in mid-cavity ROIs) increased from 41.8% to 48.8% (*p* < 0.001) in the tafamidis-naïve group, whereas it remained stable in the tafamidis-treated group (51.2% to 51.1%, *p* = 0.052)—after a follow-up of only 12 months. In comparison, global ECV increased from 50 to 67% (*p* < 0.001) in the tafamidis-naïve group B in the present study with longer follow-up, whereas global ECV also increased—however to a lesser extent—in the tafamidis-treated group A (51% to 57%, *p* < 0.001). In comparison, acoramidis resulted in a less increase in ECV (62% to 64%) even after 30 months of treatment [14]. Taken together, the data from Duca et al. [13] as well as our prior and present study data [11] clearly suggest that the TTR-stabilizer tafamidis may “stop” the ongoing process of myocardial amyloid accumulation (reflected by the CMR-based ECV value) only for approximately 12 months and that an increase in myocardial amyloid deposition occurs after a treatment period of 2 years. Hence, additional and/or more effective therapeutic options (in addition to or as an alternative for tafamidis) are required if we aim to “stop” myocardial amyloid accumulation beyond 12 months of treatment [16].

Noteworthy, we do not have comparable CMR-data regarding the use of gene-silencers (such as patisiran, vutrisiran, inotersen or eplontersen) for such a long treatment period of 2 years and beyond in ATTR-CM patients. Recent data from the Helios-B trial indicate a synergistic or even more beneficial therapeutic effect of the gene-silencer vutrisiran compared to the TTR-stabilizer tafamidis regarding hard clinical endpoints [17]. However, CMR studies were not part of the Helios-B study protocol. Prior single-center data [18] suggested a (relative) reduction in ECV of −6.2% [95%-CI −9.5% to −3.0%]; *p* = 0.001) in a small group of patisiran-treated patients compared to patisiran-naïve patients within a follow-up period of only 12 months. However, such results were not confirmed by other groups, and similar data covering a longer follow-up period of more than 12 months are not available yet.

Furthermore, based on the present study results, we may deduce some valuable numbers regarding the “natural” cardiac disease course that can be used for comparison in future clinical studies: e.g., a decrease in LV-EF of up to 10%, a decrease in RV-EF of up to 5%, an increase in LV mass of up to 15 g/m^2^, an increase in maximal LV thickness of up to 3 mm, or an increase in global ECV of up to 17%—each within approximately 2 years of follow-up.

### Long-term effect of tafamidis on serum biomarkers

Regarding longitudinal changes of cardiac biomarkers, the present results expand our knowledge regarding changes of serum NT-proBNP during the disease course of ATTR-CM. In our previous study with a follow-up time of only 12 months [11], we observed a concurrent increase in NT-proBNP serum values in both groups (with and without tafamidis treatment)—without significant differences between both groups (2,068 pg/ml to 2,403 pg/ml in the tafamidis-treated group vs. 1,810 pg/ml to 2,614 pg/ml in the tafamidis-naïve group; ∆p = 0.20). In the current study with a longer follow-up time of 27 months, we observed substantial increases in NT-proBNP levels in both groups (1,848 pg/ml to 2,202 pg/ml in the tafamidis-treated group vs. 2,250 pg/ml to 3,717 pg/ml in the tafamidis-naïve group), however, with a significantly higher increase in the tafamidis-naïve group (∆p = 0.014). Hence, tafamidis treatment seems to significantly slow down the increase in serum NT-proBNP levels (reflecting ongoing myocardial stiffening due to amyloid depositions) after a treatment period of approximately 2 years. Moreover, tafamidis-naïve patients (group B) showed a considerable and significant increase in serum troponins that was not observed in the tafamidis-treated group in the present study. Therefore, the “disease monitoring value” of troponins should be carefully compared to other biomarkers such as NT-proBNP or even imaging parameters such as ECV in future larger trials.

### Study limitations

Obviously, the gender distribution between the groups was slightly different. However, we do not expect a substantial impact on the present study results. Moreover, the sample size was not very large and this was not a prospective, randomized, controlled trial. However, a total study size of 56 patients for a proven rare disease such as ATTR-CM is not very small, and the availability of two comprehensive serial CMR studies in each patient clearly outclasses standard cardiac workup. Finally, standardised data from other disciplines and methods (e.g., neurological examination or bone scintigraphy) were not available and therefore not considered in the present analysis.

## Conclusion

Substantial worsening of clinical symptoms, serum biomarkers and imaging parameters occurs in both tafamidis-treated and tafamidis-naïve ATTR-CM patients within a follow-up period of approximately 2 years. However, the “extent of worsening” is significantly lower in tafamidis-treated compared to tafamidis-naïve patients.

## Data Availability

The datasets used and/or analyzed during the current study are available from the corresponding author on reasonable request.

## Abbreviations

ATTRwt: Wild-type transthyretin amyloidosis
ATTRv: Hereditary/variant transthyretin amyloidosis
BMI: Body mass index
CA: Cardiac amyloidosis
CM: Cardiomyopathy
CMR: Cardiovascular magnetic resonance
ECV: Extracellular volume fraction
eGFR: Estimated glomerular filtration rate
EMB: Endomyocardial biopsy
LV: Left ventricular
LV-EF: Left ventricular ejection fraction
LV-EDVi: Left ventricular end-diastolic volume index
LV-GLS: Left ventricular global longitudinal strain
LVMi: Left ventricular mass index
LVWT: Left ventricular wall thickness
LGE: Late gadolinium enhancement
NAC: National Amyloidosis Centre
NT-proBNP: N-terminal pro brain natriuretic peptides
NYHA: New York Heart Association
RV-EF: Right ventricular ejection fraction
RV-EDVi: Right ventricular end-diastolic volume index
